# Stereoacuity of Black-White and Red-Green Patterns in Individuals with and without Color Deficiency

**DOI:** 10.1155/2018/1926736

**Published:** 2018-08-01

**Authors:** Ying Sun, Huang Wu, Yinghong Qiu, Zhiqiang Yue

**Affiliations:** ^1^The First Hospital of Jilin University, Changchun, China; ^2^The Second Hospital of Jilin University, Changchun, China; ^3^Ophthalmology Hospital of Hebei Province, Hebei, China

## Abstract

**Background:**

Chromatic contrast may affect stereopsis. Daltonism is a common color deficiency in which the colors red and green are incorrectly detected. The aim of this study was to evaluate the stereoacuity of color-defective individuals presented with color symbols that they see defectively.

**Methods:**

Ten students diagnosed with daltonism and 10 students with normal color vision were recruited. A stereopsis test system using a phoropter and two 4K smartphones was used. Contour-based graphs and random-dot graphs with black versus white and red versus green patterns were used as test symbols. The Wilcoxon signed rank test was used to test the difference between groups.

**Results:**

No significant difference in stereoacuity was found between contour-based and random-dot graphs within both daltonism cohort and normal color vision cohort (*P* > 0.05). A significant difference in stereoacuity was found between the black-white (*P*=0.005) and red-green (*P*=0.007) graphs for the daltonism cohort, while no significant difference in stereoacuity was found for the normal color vision cohort (*P* > 0.05).

**Conclusion:**

Chromatic contrast is an influential factor for stereopsis measurement in individuals with color deficiency.

## 1. Background

Stereopsis facilitates the precise judgment of distance, and stereoacuity is used to evaluate it. Stereoacuity has been measured using the following tests: the Howard-Dolman test [1], the Frisby stereo test [2], the TNO (The Netherlands Optical Society) stereoacuity test [3], and the Titmus stereoacuity test [4]. With the development of information technology, the computer has become a useful tool for evaluating stereopsis, from the cathode ray tube monitor used in the 1980s [5] to the three-dimensional (3D) liquid crystal display or light-emitting diode applied after the twenty-first century [6–8] and finally to the 4K smartphone currently used [9]. The new methods facilitate improved measurement of stereopsis compared with traditional ones. For example, the relationships between chromatic contrast and stereopsis can be evaluated with a computer [5, 6], which is difficult to do using traditional methods.

Color deficiency, commonly called color blindness, is a disorder that causes people to distinguish colors abnormally. Daltonism is a common color deficiency in which people cannot detect red and green colors correctly. There is a paucity of studies investigating the change in stereoacuity when color blind individuals see symbols in the colors for which their vision is deficient. In the current study, we reevaluated 19 color-deficient freshmen who were preliminarily diagnosed at a school student health center. A newly designed stereopsis test system was used to evaluate the stereoacuity associated with a black-white or a red-green pattern. Students with and without daltonism were tested.

## 2. Methods

### 2.1. Participants

Ten students with daltonism diagnosed using a pseudoisochromatic plate test [10] and 10 students with normal color vision were recruited. The correct visual acuity of each eye was no less than 0 logMAR, while the stereoacuity was no less than 40″ as measured using the Fly Stereo Acuity Test (Vision Assessment Corporation, Elk Grove Village, IL, USA). The other 9 out of the 19 color-deficient students were excluded due to unqualifying stereopsis, or the degree of color deficiency was just red and/or green weakness.

All participants gave their informed written consent before taking part in the study. The research protocol observed the tenets of the Declaration of Helsinki and was approved by the Ethics Committee of the Second Hospital of Jilin University (no. 2017-89).

### 2.2. Test Equipment

**Figure 1:**
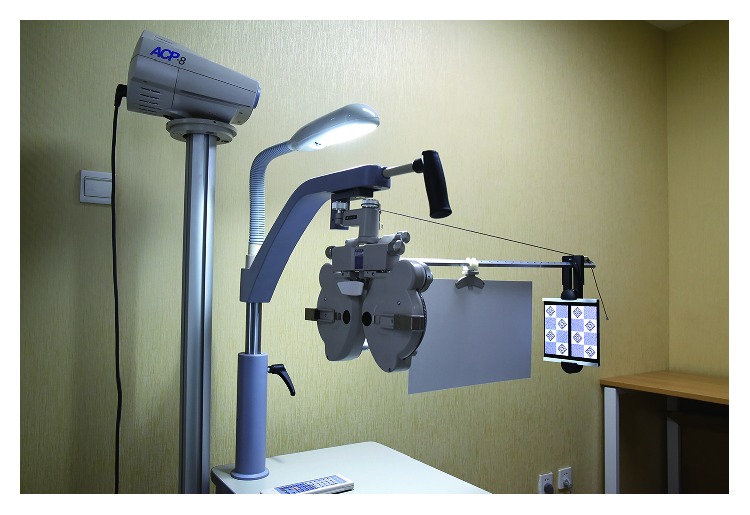
The test system used consisted of a phoropter and two Sony smartphones. A pair of smartphone pictures are seen with a black and white pattern.

We incorporated a stereopsis measurement system using a phoropter (Topcon VT-10; Topcon Corp., Tokyo, Japan) and two Sony smartphones (Sony Xperia Z5 Premium Dual E6883; resolution, 3840 × 2160; Sony Mobile Communications Inc., Tokyo, Japan) [9]. The test distance was 65 cm. One pixel disparity represents 10″ (acrsec) at this distance. With the aid of two 5.5Δ base-out Risley prisms, the subject can fuse the two smartphones into one image (Figure 1). A screen luminance meter (SM208; M&A Instrument Inc., Shenzhen, China) was used to measure the brightness of the display. A program was written using C# to generate all random-dot stereograms. Crossed disparity was used in all test graphs.

### 2.3. Test Symbols

**Figure 2:**
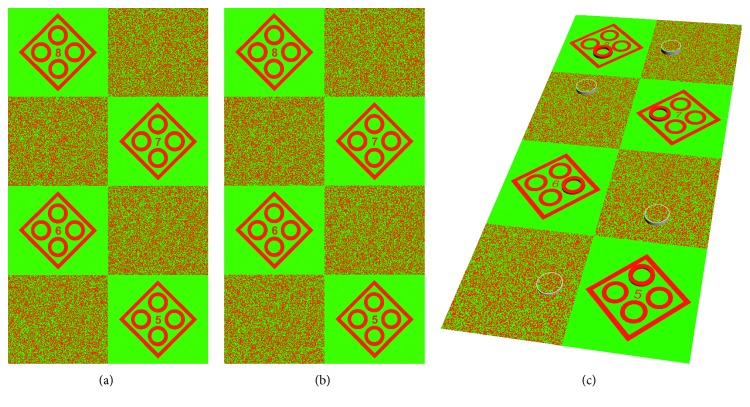
Test pages used. A pair with a red and green pattern seen by the (a) left eye and (b) right eye. From top to bottom, the disparity of the stereo targets is 80″, 70″, 60″, and 50″, respectively. From top to end, the stereo symbol in contour-based graphs is left, right, down, and up, respectively, while the stereo symbol in random-dot graphs is down, up, right, and left, respectively. (c) The simulation of the percepts generated by the test images (a, b). This is an attempt to simulate what a subject might perceive when fusing (a) and (b) as one image. The stereo symbols appear to pop out of the background plane.

Two types of symbols, a contour-based graph and a random-dot graph, were used (Figure 2). The shape of the contour-based symbol was similar to that used in the Fly Stereo Acuity Test. One stereo circle stands out from the other three circles if the stereopsis threshold of the subject is better than the disparity of the target circle. The shape of the random-dot symbol was also similar to that used in the Fly Stereo Acuity Test. A circle appears up, down, right, or left in the random-dot graph when the disparity of the stereo target is larger than the stereoacuity of the participant. Eight different groups of disparities were drawn from 80″ to 10″. One test page contained 80″ to 50″, and the other contained 40″ to 10″.

Two types of test pages were used, black versus white and red versus green. The RGB (red, green, and blue) codes of the black, white, red, and green colors used were (*R* = 0, *G* = 0, *B* = 0), (*R* = 255, *G* = 255, *B* = 255), (*R* = 255, *G* = 0, *B* = 0), and (*R* = 0, *G* = 255, *B* = 0), respectively.

### 2.4. Test Procedure

The sequence of test pages presented was a black-white pattern with 80″ to 50″, a black-white pattern with 40″ to 10″, a red-green pattern with 80″ to 50″, and a red-green pattern with 40″ to 10″. The participants pointed out the position of the outstanding circle in the contour-based and random-dot tests, line by line from left to right and from top to bottom, until they could not find the stereo one. The disparities of the last correct identification were recorded as their stereoacuity.

### 2.5. Statistical Analysis

All data were analyzed using the PASW Statistics 18 software (IBM SPSS Inc., Chicago, IL). The Wilcoxon signed rank test was used to test the difference between groups.

## 3. Results

**Figure 3:**
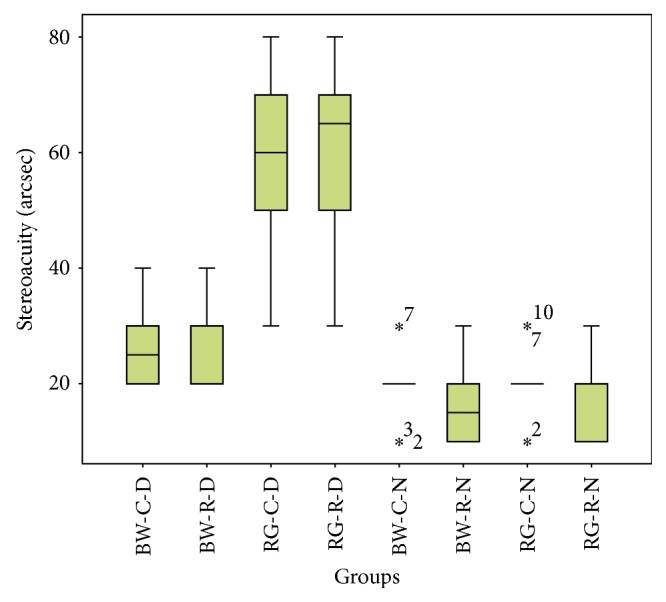
Boxplot of the stereoacuity of the following groups: BW-C-D: daltonism cohort tested with black-white contour-based graphs; BW-R-D: daltonism cohort tested with black-white random-dot graphs; RG-C-D: daltonism cohort tested with red-green contour-based graphs; RG-R-D: daltonism cohort tested with red-green random-dot graphs; BW-C-N: normal color vision cohort tested with black-white contour-based graphs; BW-R-N: normal color vision cohort tested with black-white random-dot graphs; RG-C-N: normal color vision cohort tested with red-green contour-based graphs; RG-R-N: normal color vision cohort tested with red-green random-dot graphs. The line perpendicular to the whisker below the box represents the minimum value; the lower edge of the box represents the first quartile; the line in the box is the median; the upper edge of the box represents the third quartile; and the line perpendicular to the whisker above the box represents the maximum value. The stars represent extreme values.

The test results are shown in Table 1 and Figure 3. No significant differences were found between the results for contour-based and random-dot graphs within the cohorts with and without daltonism (Wilcoxon signed rank test: a black-white pattern in the daltonism group: *Z*=−1.000, *P*=0.317; a red-green pattern in the daltonism group: *Z*=−1.414, *P*=0.157; a black-white pattern in the normal group: *Z*=−1.732, *P*=0.083; and a red-green pattern in the normal group: *Z*=−1.342, *P*=0.180). A significant difference was found between the results for black-white and red-green test pages in the cohort with daltonism (Wilcoxon signed rank test: contour-based group: *Z*=−2.814, *P*=0.005; random-dot group: *Z*=−2.714, *P*=0.007). No significant difference was found between the results for the black-white and red-green graphs in the cohort without daltonism (Wilcoxon signed rank test: contour-based group: *Z*=−1.414, *P*=0.157; random-dot group: *Z*=−1.000, *P*=0.317).

## 4. Discussion

The relationship between chromatic information and stereopsis has been studied for people with normal color vision [10, 11], although questions still exist. The mechanism of color deficiency, also a conundrum, is still only a hypothesis [12]. People with daltonism can distinguish the difference between red and green, but see red and green differently than people with normal color vision. Chromatic symbols for red and green test pages were used; however, they were not complementary (the complementary color of red (*R* = 255, *G* = 0, *B* = 0) is blue (*R* = 0, *G* = 255, *B* = 255), and the complementary color of green (*R* = 0, *G* = 255, *B* = 0) is magenta (*R* = 255, *G* = 0, *B* = 255)). In both contour-based graphs and random-dot graphs, the contrast of the colors together with the luminant contrast of the symbols against the background (red: luminance = 48 cd/m^2^; green: luminance = 146 cd/m^2^; Weber contrast = (*I* *−* *I*_*b*_)/*I*_*b*_ = 67%) was obvious enough to keep the stereopsis level from decreasing in people with normal color vision. The situation was different for people with daltonism when observing a red-green pair graph. The luminance contrast of the symbols against the background still existed, but the comparison of the colors changed. However, it is hard to interpret why the stereoacuity measured with the red-green pair was significantly lower than that with the black-white pair in the daltonism cohort due to the decrease of color comparison. The luminant contrast in the test was not low enough to affect the stereoacuity result significantly [13]. The positive effect of chromatic contrast for stereopsis evaluation was reported in normal individuals [10, 11].

The literature about binocular vision related to color vision deficiency is rare. Bak et al. evaluated the Worth four-dot test in patients with congenital red-green color vision defects [14]. The red/green anaglyph glasses play an essential role in the Worth four-dot test, and the function of it is to dissociate right and left eyes. So no matter the normal color vision people or red-green color deficiency people, the separate function of the glasses is the same. That is, to evaluate flat fusion, the Worth four-dot test works for both normal color vision and abnormal cohorts. Furthermore, it could be speculated that if using red/green anaglyph glasses as a dissociation tool to evaluate stereopsis, that is, TNO, although no literature be retrieved, it may still work for red-green color vision defects people. If the color elements were not used as a way to separate eyes, but as constituent parts in the test patterns, the situation would change. In our experiment, the color information used in the experiment did not enhance but rather interfered with the stereopsis in individuals with color-defective vision. The difference in stereoacuity between people with normal color vision and people with daltonism when adding chromatic information has not been reported.

The limitations of our research were the small size of samples and the diagnosing method was not conducted with more quantitative tools, that is, anomaloscope. However, future studies need to be designed to determine the etiology related to why people see color information achromatically in the procedure for measuring stereopsis.

## 5. Conclusion

The stereoacuity evaluated with red-green and black-white symbols was significantly different for people with daltonism and not significantly different for people with normal color vision. Chromatic contrast influences the stereopsis measurement.

## Figures and Tables

**Table 1:** Stereoacuity (″) of the participants.

ID	Daltonism				Normal			
	Black versus white		Red versus green		Black versus white		Red versus green	
	Contour-based	Random-dot	Contour-based	Random-dot	Contour-based	Random-dot	Contour-based	Random-dot
1	20	20	60	60	20	10	20	20
2	20	20	30	30	10	10	10	10
3	30	40	60	70	10	10	20	10
4	30	30	50	50	20	10	20	20
5	20	30	70	70	20	20	20	20
6	40	40	80	80	20	20	30	20
7	20	20	80	80	20	30	30	20
8	20	30	30	30	20	10	20	10
9	30	20	60	60	20	20	20	30
10	30	30	60	70	20	20	30	20

## Data Availability

The data used to support the findings of this study are available from the corresponding author upon request.

## References

[1] Saladin J. J., Benjamin W. J. (2006). Phorometry and stereopsis. *Borish’s Clinical Refraction*.

[2] Bohr I., Read J. C. (2013). Stereoacuity with Frisby and revised FD2 stereo tests. *PLoS One*.

[3] van Doorn L. L., Evans B. J., Edgar D.F., Fortuin M. F. (2014). Manufacturer changes lead to clinically important differences between two editions of the TNO stereotest. *Ophthalmic and Physiological Optics*.

[4] Arnoldi K., Frenkel A. (2014). Modification of the titmus fly test to improve accuracy. *American Orthoptic Journal*.

[5] Shoji K., Sumi S., Fujita H. (1980). Depth perception in moving line patterns. *Perceptual and Motor Skills*.

[6] Han S. B., Yang H. K., Kim J., Hong K., Lee B., Hwang J. M. (2015). New stereoacuity test using a 3-dimensional display system in children. *PLoS One*.

[7] Kim J., Yang H. K., Kim Y., Lee B., Hwang J. M. (2011). Distance stereotest using a 3-dimensional monitor for adult subjects. *American Journal of Ophthalmology*.

[8] Wu H., Jin H., Sun Y. (2016). Evaluating stereoacuity with 3D shutter glasses technology. *BMC Ophthalmology*.

[9] Wu H., Liu S., Wang R. (2017). Stereoacuity measurement using a phoropter combined with two 4K smartphones. *Clinical and Experimental Optometry*.

[10] Kingdom F. A., Simmons D. R. (1996). Stereoacuity and colour contrast. *Vision Research*.

[11] Simmons D. R., Kingdom F. A. (2002). Interactions between chromatic- and luminance-contrast-sensitive stereopsis mechanisms. *Vision Research*.

[12] Pease P. J., Benjamin W. J. (2006). Color vision. *Borish’s Clinical Refraction*.

[13] Halpern D. L., Blake R. R. (1988). How contrast affects stereoacuity. *Perception*.

[14] Bak E., Yang H. K., Hwang J. M. (2017). Validity of the Worth 4 dot test in patients with red-green color vision defect. *Optometry and Vision Science*.

